# Sequential Episodes of Dengue—Puerto Rico, 2005–2010

**DOI:** 10.4269/ajtmh.13-0742

**Published:** 2014-08-06

**Authors:** Tyler M. Sharp, Elizabeth Hunsperger, Jorge L. Muñoz-Jordán, Harold S. Margolis, Kay M. Tomashek

**Affiliations:** Epidemic Intelligence Service, Centers for Disease Control and Prevention, Atlanta, Georgia; Dengue Branch, Division of Vector-Borne Diseases, Centers for Disease Control and Prevention, San Juan, Puerto Rico

## Abstract

Of 53,633 suspected dengue cases reported to a passive dengue surveillance system in Puerto Rico during 2005–2010, 949 individuals were reported on more than one occasion and 21 had laboratory-confirmed dengue on two separate occasions. Median time between illness episodes was 2.9 years (range: 62 days–5.3 years). Seventeen (81%) individuals with sequential episodes of dengue were male, and seven (33%) were adults. All 21 individuals experienced one episode and seven (33%) individuals experienced both episodes during a large epidemic that occurred in 2010. These observations show that heterotypic dengue virus immunity that protects against illness may have considerable variability but typically does not last longer than 3 years.

Dengue is the most important vector-borne viral disease globally,^1^ and an estimated 96 million cases occurred in 2010.^2^ Dengue is caused by infection with any of four dengue virus-types (DENV-1–4) that are transmitted by some *Aedes* species mosquitoes.^1^ Although most DENV infections are asymptomatic or subclinical,^3^ ∼5% of dengue cases develop potentially fatal severe dengue, characterized by vascular leakage and hypovolemic shock.^1^

Following six outbreaks of dengue-like illness in Galveston, Texas between 1897 and 1922, Sharp and Hollar estimated that dengue immunity lasts between 1 and 4 years.^4^ Later studies by Sabin involving experimental infection of humans with different DENV-types suggested that cross-protective (i.e., heterotypic) immunity protects from illness for 2 months and attenuates illness for up to 9 months.^5^ Contemporary studies of naturally occurring infections in children suggest that heterotypic immunity protects against illness for 1–3 years,^6^–^9^ and homotypic immunity is long-lived and specific to the infecting DENV-type.^10^

The U.S. Centers for Disease Control and Prevention Dengue Branch (CDC-DB) and Puerto Rico Department of Health (PRDH) have operated the island-wide passive dengue surveillance system (PDSS) for several decades,^11^ which most recently detected epidemics in 2007^12^ and 2010^13^ (Figure 1) that were caused primarily by DENV-2 and -3, and DENV-1 and -4, respectively. The objective of this study was to use a retrospective, descriptive case series to characterize the epidemiology and DENV immunologic profile of individuals found to have sequential episodes of dengue as detected by PDSS. This study was approved by the Institutional Review Boards at CDC (protocol no. 6383).

**Figure 1.**
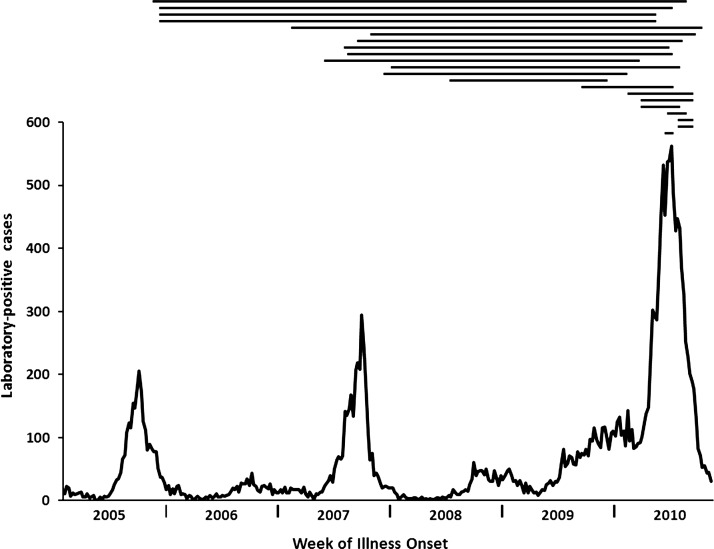
Time between illness onset dates for individuals experiencing sequential episodes of dengue in Puerto Rico, 2005–2010 (*N* = 21). Plotted on the *y* axis is the number of reported, laboratory-positive dengue cases each week between 2005 and 2010. Each horizontal black line represents one individual that experienced sequential episodes of dengue. The start point of each line indicates the onset date of the first reported illness, and the end point indicates the onset date of the second reported illness. Lines are ordered vertically by length.

Dengue is a reportable health condition in Puerto Rico, and although the majority of laboratory-positive cases are identified by PDSS each year,^13^ the current magnitude of underreporting of suspected cases is unclear. To report a suspected dengue case to PDSS, a clinician sends the patient's serum specimen along with a Dengue Case Investigation Form (DCIF) (available at www.cdc.gov/dengue/resources/dengueCaseReports/DCIF_English.pdf) to CDC-DB. Following dengue diagnostic testing of specimens, all data are entered into a secure database, results are reported to the referring clinicians, and epidemiologic trends are analyzed. Unique identifiers are used to identify both reported individuals and their illness episodes. Cases reported with illness onset dates < 14 days apart and matching names and dates of birth are consolidated into a single case.

All serum specimens from suspected dengue cases were tested as previously described.^12^,^13^ Briefly, specimens collected within 4 days of illness onset were tested by DENV-type specific, multiplex reverse transcriptase-polymerase chain reaction (RT-PCR),^14^ and specimens collected 6 days or more after illness onset were tested by anti-DENV immunoglobulin M (IgM) antibody by capture enzyme-linked immunosorbent assay (MAC ELISA).^15^ From 2005 to 2006, specimens collected 5 days after illness onset were tested only by RT-PCR. From 2007 to 2010, specimens collected 5 days after illness onset were tested by both RT-PCR and MAC ELISA. Laboratory-positive dengue cases were defined either by detection of DENV nucleic acid by RT-PCR or anti-DENV IgM antibody by MAC ELISA in a serum specimen. Neutralizing antibody profiles were determined by microneutralization (MNT) assay^16^ for all available serum specimens. Due to the possibility of original antigenic sin,^17^ if an individual's illness was defined solely by the presence of anti-DENV IgM antibody, the infecting DENV-type was defined solely by a monotypic immune response in which a MNT titer was present against a single DENV-type.

To identify individuals defined as a laboratory-positive dengue case on multiple occasions, the PDSS database was queried for cases with identical dates of birth and first and last names. Fourteen individuals with a second illness that was positive solely by MAC ELISA ≤ 90 days after a previous laboratory-confirmed episode of dengue were excluded from further analysis because anti-DENV IgM antibody can in some cases be detected up to 90 days after illness onset.^18^ Also excluded from further analysis were two individuals with DENV-1 and -4 detected in a single specimen in 2010, and one individual with DENV-2 detected by RT-PCR during independent illness episodes 16 months apart because virus could not be isolated from the specimen collected during the first illness.

Of 53,633 suspected dengue cases reported to PDSS between January 1, 2005 and December 31, 2010, 949 individuals were reported on more than one occasion (902 twice, 33 three times, 12 four times, and 2 five times). Of 20,867 laboratory-positive cases, 21 (0.1%) were individuals that had dengue on at least two occasions (Table 1). Four (19%) of these individuals (case ID no. 3, 4, 5, and 10) had serologic evidence of infection with a DENV before the first documented episode, suggesting that they were experiencing tertiary or quaternary DENV infection on their second documented episode of dengue.

Seventeen (81%) individuals with sequential episodes of dengue were male (relative risk ratio [RR] = 3.5, 95% confidence interval [95% CI]: 1.2–10.3). Median age during the first episode was 15 years (range: 0.5–73). Seven (33%) individuals were adults, which was not significantly different from all laboratory-positive dengue cases (54%; RR = 0.4, 95% CI: 0.2–1.1). Median time between symptom onset dates was 2.9 years (range: 62 days–5.3 years). Eight (38%) of the 21 identified individuals experienced the first episode in 2007, all (100%) experienced the second episode during the 2010 epidemic, and seven (33%) experienced both episodes during the 2010 epidemic (Figure 1).

Eight (38%) individuals were defined as laboratory-positive dengue cases by RT-PCR during both episodes, nine (43%) by MAC ELISA during the first episode and RT-PCR during the second, three (14%) by RT-PCR during the first episode and MAC-ELISA during the second, and one (5%) by MAC-ELISA during both episodes. Both infecting DENV-types were identified by RT-PCR or inferred by neutralizing antibody patterns for 10 individuals, of which five (50%) were DENV-3 followed by DENV-4, three (30%) were DENV-1 followed by DENV-4, and one (10%) each was DENV-3 followed by DENV-1 and DENV-2 followed by DENV-1.

The duration of heterotypic DENV immunity that protects against illness has recently been estimated to have an average duration of 1 to 3 years based on 8 years of data collected from a prospective pediatric cohort in Nicaragua,^9^ modeling of 38 years of dengue surveillance data from Thailand,^6^ and a study that used data from two prospective pediatric cohorts from Thailand involving 2,169 person-years of observation.^8^ Interestingly, increased time between infections was associated with increased disease severity in both the Thailand and Nicaragua cohorts.^8^,^9^ In addition, 11 years of surveillance data from Thailand identified 191 individuals with repeat hospital admission for laboratory-confirmed dengue, for which the median interval between dengue episodes was 3.5 years and ranged from 4 months to 9 years.^7^ Finally, a study from Puerto Rico based on at least 13 years of PDSS data showed that of nine individuals experiencing sequential episodes of dengue, the median time between episodes was 4 years and ranged from < 2 to 13 years.^19^ The present study observed a median interval of nearly 3 years in 21 individuals with sequential episodes of dengue, and most episodes occurred during an epidemic in 2010 that followed a shift in the dominate DENV-types.^12^,^13^,^20^ These six studies were all differentially affected by the occurrence of epidemics and consequent differences in force of infection; circulating DENV-types and the order in which infections occurred, which may affect disease severity^21^; and an inability to accurately determine when individuals became susceptible to reinfection. Nonetheless, they are all in general agreement that on average heterotypic DENV immunity does not protect against illness for more than 3 years.

Because males consistently represent half of all DENV infections in Puerto Rico,^12^,^13^ an unexpected finding of this study was that males were more than three times more likely than females to experience sequential episodes of dengue. Similarly, a previous study from Puerto Rico reported that males represent two-thirds of identified individuals with sequential episodes of dengue.^19^ Moreover, although both symptomatic and inapparent infections were equally distributed by gender in a Nicaraguan pediatric cohort, repeat DENV infections appeared to be more frequent in males; however although this difference only approached statistical significance.^9^ Taken together, the findings of these three studies provide weak evidence for a sex-specific difference in the duration of protective heterotypic DENV immunity. Previous studies of other pathogens have reported that males have increased susceptibility to infection^22^ and development of disease,^23^–^25^ which may be attributable in part to the effect of estrogen.^24^,^26^ Additional studies with a larger population of individuals that experienced sequential episodes of dengue are therefore needed to confirm the finding of sex-specific differences in the duration of protective heterotypic immunity and elucidate potential underlying mechanisms.

As PDSS is more likely to capture severe rather than mild dengue cases and was previously estimated to have a 10- to 27-fold rate of underreporting,^27^,^28^ these surveillance biases likely led to underestimation of the number of individuals that experienced sequential episodes of dengue and potential misrepresentation of the median and minimal period of time between episodes. In addition, the rate of sequential episodes of dengue observed herein was 0.1% of all reported clinically apparent dengue cases, which is noticeably less than the 1.2%^7^ to 2%^9^ observed in prior studies that used data gathered through hospital-based surveillance and a prospective cohort study, respectively. Similarly, the rate of tertiary or quaternary DENV infection identified in this study was 0.02% of all identified dengue cases, compared with a previous observation of 0.08%.^7^

A prominent strength of this study was use of surveillance data from both pediatric and adult health care settings to identify adults as accounting for one-third of all individuals with sequential episodes of dengue. Because most, but not all,^5^,^19^ previous studies of heterotypic DENV immunity used data gathered through surveillance and cohort studies conducted in children, future studies should include adults to identify potential age-specific differences in the duration of protective heterotypic immunity. A limitation of this study was that dengue surveillance in Puerto Rico by definition does not collect data on asymptomatic DENV infections. Consequently, although we were able to estimate the rate of sequential dengue episodes over the time frame of this study, we were unable to estimate the rate of sequential DENV infections. Similarly, inconsistent reporting of clinical data to PDSS and unavailability of patients' medical records precluded analysis of the association of time between infections and disease severity.

In conclusion, we identified 21 individuals that had sequential episodes of dengue in Puerto Rico between 2005 and 2010. The minimal period of time between episodes was 62 days, indicating that heterotypic immunity that is protective against illness can in some cases be quite short-lived, but was most often < 3 years. This study shows that potential age and sex-specific differences in protective heterotypic immunity should be further investigated, specifically in relation to the dosing regimen of a multivalent dengue vaccine.

## Figures and Tables

**Table 1:** Laboratory characteristics of individuals experiencing sequential episodes of dengue, Puerto Rico, 2005–2010*

ID no.	Sex	Age at first illness onset (years)	First identified episode of dengue							Days between illness onset dates	Second identified episode of dengue						
			Diagnostic test	DPO	MNT titer				Infecting DENV		Diagnostic test	DPO	MNT titer				Infecting DENV
					DENV-1	DENV-2	DENV-3	DENV-4					DENV-1	DENV-2	DENV-3	DENV-4	
1	M	22	MAC ELISA	5					UNK	62	RT-PCR	3					DENV-1
2	M	35	RT-PCR	4					DENV-2	94	MAC ELISA	5					UNK
3	M	73	RT-PCR	1	1,280	> 2,560	1,280	640	DENV-4	103	MAC ELISA	6	2,560	10,240	40,960	5,120	UNK
4	M	1	RT-PCR	0	2,560	1,280	1,280	2,560	DENV-1	120	RT-PCR	3					DENV-4
5	M	16	RT-PCR	3	160	< 40	> 2,560	80	DENV-1	161	RT-PCR	5					DENV-4
6	M	42	RT-PCR	1	> 2,560	< 40	160	80	DENV-1	301	RT-PCR	3	80	320	640	80	DENV-4
7	M	12	MAC ELISA	6					UNK	353	RT-PCR	3					DENV-4
8	F	27	MAC ELISA	6					UNK	407	RT-PCR	4					DENV-4
9	M	6	MAC ELISA	4	< 40	< 40	5,120	< 40	DENV-3†	728	RT-PCR	2	640	5,120	20,480	2,560	DENV-4
10	M	13	RT-PCR	1	> 2,560	> 2,560	> 2,560	> 2,560	DENV-3	810	RT-PCR	3	320	640	80	640	DENV-1
11	M	15	MAC ELISA	6	< 40	< 40	2,560	< 40	DENV-3†	1,056	RT-PCR	2	1,280	20,480	40,960	2,560	DENV-4
12	F	0.6	RT-PCR	3					DENV-2	1,060	RT-PCR	2					DENV-1
13	M	12	RT-PCR	3					DENV-3	1,066	RT-PCR	4	> 2,560	> 2,560	> 2,560	> 2,560	DENV-4
14	M	12	MAC ELISA	6					UNK	1,122	RT-PCR	3	1,280	640	> 2,560	2,560	DENV-4
15	F	15	RT-PCR	3					DENV-3	1,143	RT-PCR	3	160	1,280	> 2,560	> 2,560	DENV-4
16	M	6	RT-PCR	3	320	< 40	320	80	DENV-3	1,241	RT-PCR	3	640	320	5,120	2,560	DENV-4
17	M	17	RT-PCR	1					DENV-3	1,405	MAC ELISA	7					UNK
18	M	0.5	MAC ELISA	5					UNK	1,717	MAC ELISA	10					UNK
19	M	18	MAC ELISA	7					UNK	1,754	RT-PCR	4	> 2,560	> 2,560	> 2,560	> 2,560	DENV-1
20	F	46	MAC ELISA	8	1,280	20,480	5,120	1,280	DENV-2	1,853	RT-PCR	3	2,560	1,280	2,560	1,280	DENV-4
21	M	1	MAC ELISA	4					UNK	1,935	RT-PCR	4	160	5,120	2,560	320	DENV-1

* Serum specimens from individuals reported as a suspected dengue case were tested for evidence of DENV infection. Microneutralization antibody titers were determined for all available specimens.

† Inferred by neutralizing antibody patterns.

DPO = day post-illness onset specimen was taken; DENV = dengue virus; MNT = microneutralization assay; M = male; F = female; MAC-ELISA = anti-DENV IgM antibody-capture enzyme-linked immunosorbent assay; RT-PCR = DENV-type-specific reverse transcription polymerase chain reaction; UNK = unknown.
